# Root-Associated Endophytic Bacterial Community Composition of *Pennisetum sinese* from Four Representative Provinces in China

**DOI:** 10.3390/microorganisms7020047

**Published:** 2019-02-11

**Authors:** Zhen-Shan Deng, Bao-Cheng Zhang, Xiang-Ying Qi, Zhi-Hong Sun, Xiao-Long He, Yu-Zhen Liu, Jing Li, Kai-Kai Chen, Zhan-Xi Lin

**Affiliations:** 1 College of Life Sciences, Yan’an University, Yan’an 716000, China; yd_qixiangying@163.com (X.-Y.Q.); sunzhihong1968@163.com (Z.-H.S.); ydhelong@163.com (X.-L.H.); 18291165056@163.com (Y.-Z.L.); lijing@126.com (J.L.); chenkk@126.com (K.-K.C.); 2 School of Biological and Agricultural Science and Technology, Zunyi Normal College, Zunyi 53602, China; woshimiantian@126.com; 3 National Engineering Research Center of Juncao, Fuzhou 350002, China; linzhanxi@163.com

**Keywords:** endophytic bacteria, high-throughput sequencing, bacterial community composition, *Pennisetum sinese*

## Abstract

*Pennisetum sinese*, a source of bio-energy with high biomass production, is a species that contains high crude protein and will be useful for solving the shortage of forage grass after the implementation of “Green for Grain” project in the Loess plateau of Northern Shaanxi in 1999. Plants may receive benefits from endophytic bacteria, such as the enhancement of plant growth or the reduction of plant stress. However, the composition of the endophytic bacterial community associated with the roots of *P. sinese* is poorly elucidated. In this study, *P. sinese* from five different samples (Shaanxi province, SX; Fujian province, FJ; the Xinjiang Uyghur autonomous prefecture, XJ and Inner Mongolia, including sand (NS) and saline-alkali land (NY), China) were investigated by high-throughput next-generation sequencing of the 16S rDNA V3-V4 hypervariable region of endophytic bacteria. A total of 313,044 effective sequences were obtained by sequencing five different samples, and 957 effective operational taxonomic units (OTUs) were yielded at 97% identity. The phylum Proteobacteria, the classes Gammaproteobacteria and Alphaproteobacteria, and the genera *Pantoea*, *Pseudomonas*, *Burkholderia*, *Arthrobacter*, *Psychrobacter*, and *Neokomagataea* were significantly dominant in the five samples. In addition, our results demonstrated that the Shaanxi province (SX) sample had the highest Shannon index values (3.795). We found that the SX (308.097) and NS (126.240) samples had the highest and lowest Chao1 richness estimator (Chao1) values, respectively. Venn graphs indicated that the five samples shared 39 common OTUs. Moreover, according to results of the canonical correlation analysis (CCA), soil total carbon, total nitrogen, effective phosphorus, and pH were the major contributing factors to the difference in the overall composition of the bacteria community in this study. Our data provide insights into the endophytic bacteria community composition and structure of roots associated with *P. sinese.* These results might be useful for growth promotion in different samples, and some of the strains may have the potential to improve plant production in future studies.

## 1. Introduction

In both natural and anthropic ecosystems, plants interact with a wide range of microorganisms, including bacteria. Recently, authors in [1] described endophytes as “all microorganisms which for all or part of their lifetime colonize internal plant tissues without causing any damage or disease to the host plants.” Endophytic bacteria have been isolated from different plant tissues, such as roots, stems, leaves, flowers, and seeds [2,3].

Endophytic bacteria live inside a plant for at least part of their life cycle and do not visibly harm host plants. The host plant provides endophytes with a supply of nutrients and shelter from most abiotic stresses. In return, plants may receive benefits from microbial associations, such as the enhancement of plant growth or reduction of plant stress, e.g., through growth promotion, pathogen suppression, contaminant removal, phosphate solubilization, nitrogen fixation, etc. [4,5,6,7,8,9].

*Pennisetum sinese* is a hybrid of *Pennisetum purpureum* and *Pennisetum americanum*, and it is a monocot C_4_ perennial grass that is used as a bioenergy source. It is tall and forms in robust bamboo-like clumps. This species has high biomass production, at about 40 t ha^−1^year^−1^, and it can be harvested 3–4 times per year. *P. sinese* is a versatile and adaptable plant. It can grow and even thrive in many weather conditions, growing seasons lengths, soil types, and land conditions. The crude protein content of *P. sinese* is approximately 15–22% [10]. The Loess Plateau of China, characterized by extensive wind-blown sedimentary deposits, is one of the most eroded areas in the world due to its naturally erodible soil. In response to the environmental crisis, from 1999, China has launched the “Grain for Green” project, which aimed to convert farmland to forest, shrub and grassland. Meantime, the mode of animal husbandry and breeding has changed from the traditional “wild environment” to the “captive”. Nevertheless, with the implementation of “Green for Grain” action, a shortage of forage grass has emerged, which was the major constraints on development of animal husbandry in the Loess plateau of Northern Shaanxi. Due to high crude protein and sugar contents, fast growth, a strong tillering ability, a high yield of grass, strong resistance to drought, and good nutritional value and palatability, the utilization of *P. sinese* as the primary source of forage is promising for the Loess plateau of Northern Shaanxi. Moreover, *P. sinese* has become a new method to promote the development of local animal husbandry in recent years. Therefore, *P. sinese* was selected for this study because of its high annual biomass yield.

Although several investigations have already revealed many important aspects of the biology, physiology, and ecology of *P. sinese* [10,11,12], little systemic observation has been paid to the real composition and role of bacterial endophytes in this plant.

Culture-based detection methods are time-consuming and complex, and the state of microorganisms in some non-culturable and viable but non-culturable (VBNC) organisms may be ignored [13]. However, as many endophytic microbes are uncultivatable, the vast majority of endophytic microbial species associated with *P. sinese* are still unknown. Culture-independent molecular approaches are frequently used to characterize the compositions and structures of bacterial communities because they are time-efficient and labor-saving [14].

In addition, many reports indicate that endophytic bacteria diversity could extend our knowledge of bacteria–plant interactions, which could be used for improving host plant production and quality [15,16]. Nowadays, high-throughput sequencing (HTS) technology can be used to characterize the microbial community composition of complex environmental ecosystems. HTS technology has substantially increased our understanding of microbial population diversities in the environment and allows for the accurate identification of microbial taxa. The HTS technique of next-generation sequencing is a more robust microbial characterization technique compared to conventional culturing methods.

In the case of *P. sinese*, little information exists about the actual composition of the endophytic communities in *P. sinese*. On the other hand*,* which factors shape community structure within individual host plants remains unclear. Therefore, in this study, *P. sinese* from five different samples (Shaanxi province, Fujian province, Xinjiang, and Inner Mongolia (in sand and saline-alkali soil), China) were sampled for endophytic bacteria diversity detection using advanced DNA-based molecular techniques. The ultimate objective of this study was to determine the endophytic bacteria diversity and communities in *P. sinese* from different origins and to elucidate the influences of soil and conditions at distinct sites on the composition of the endophytic bacterial community associated with the roots of *P. sinese*. Specifically, we tried to address the following questions: (1) How does the composition of the endophytic bacterial community change in response to the different regions associated with a single host? (2) What are the major factors that shape community structure?

## 2. Materials and Methods

### 2.1. Sample Collection

The roots of *P. sinese* were collected from August to October in 2018 from specimens growing at five distinct sites in four eco-regions (>400 km apart) in China; the geographic locations of the sites are presented in Table 1. Sites were chosen based on their different bioclimatic conditions. To ensure that the experiment was representative, we randomly selected five plants from each geographic location at the same growth phase, and undamaged, healthy roots were sampled in the field. For example, we first selected five plants randomly in this position according to the five-point sampling method. All samples were cut down with sterile scissors. Then, we collected upper, middle, and lower root equivalents from one plant and mixed them for a total weight of 300 g. We placed them in a sterile plastic bag and transported them to the lab, and they were processed within 24 h. The materials from the other regions and altitudes were also collected as described above. Meanwhile, in order to remove other microbial interference on the surface of roots, a surface sterilization procedure was conducted: roots from the plants were carefully rinsed free of soil under running water and then wiped off with filter paper and surface-sterilized by immersion in 95% ethanol for 30 s, then in 5% sodium hypochlorite for 5 min, and finally rinsed eight times with sterile distilled water. To confirm that the surface sterilization process was successful, the surface sterilized nodules were rolled on a potato dextrose agar (PDA) medium containing (in grams per liter) potato, 200, glucose, 20, and agar, 18. The aliquots of the sterile distilled water from the final rinse solutions were plated onto PDA plates as controls to detect possible contaminants. Roots without growth on the control plates were considered to be effectively surface-sterilized. All samples were immediately put on ice and then stored at −80 °C as soon as possible until total DNA extraction.

The physicochemical characteristics of soil samples from the collection sites were analyzed for their chemical composition according to the procedure described by the USDA (1996) [17]. The altitudes and geographical coordinates of the sampling sites were determined (Table 1).

### 2.2. Genomic DNA Extraction and PCR Amplification

All five root samples from the same site were pooled as one sample and mixed thoroughly. Approximately 300 g of roots were used for each individual DNA extraction. Finally, six samples were generated for genomic DNA extraction. Genomic DNA was extracted by DNA quick plant system kit (Tiangen, China) after maceration in liquid nitrogen following the manufacturer’s instructions. After extraction, DNA concentration and purity were determined using 1% agarose gel electrophoresis. According to the concentration, each DNA sample was diluted to a final concentration of 1 ng/µL using sterile distilled water and was then used as a DNA template. PCR amplification of the 16S rDNA V3+V4 region was conducted. PCR experiments were performed with Phusion* High-Fidelity PCR master mix with GC buffer (New England Biolabs) to ensure amplification efficiency and accuracy, and this process was run in an Eppendorf Gradient Thermocycler (Brinkman Instruments, Westbury, NY). Using diluted genomic DNA as the template, the 16S rDNA V3+V4 region was amplified with the specific primers 341F(5′-CCTAYGGGRBGCASCAG-3′) 3 and 806R (5′-GCCAATGGACTACHVGGGTWTCTAAT-3′) with the barcode [18,19].

### 2.3. Library Construction and Sequencing

Following the above amplification, the PCR products were mixed with the same volume of 1× loading buffer (containing SYB green), and the PCR amplicons were detected using 2% agarose gel electrophoresis. After that, all of the amplicons were pooled in equimolar ratios into a single tube. Then, the target sequences were extracted using a Qiagen Gel Extraction Kit (Qiagen, Germany). The libraries were constructed using a TruSeq^®^DNA PCR-Free Sample Preparation Kit (Illumina, USA), following the manufacturer’s recommendations, and index codes were added. The library quality was assessed on the Qubit^®^ 2.0 Fluorometer (Thermo Scientific) and Agilent Bioanalyzer 2100 system. At last, the library was sequenced using the Illumina HiSeq 2500 platform, and 250 bp paired-end reads were generated.

### 2.4. Statistical Analysis

To perform an accurate taxonomic assignment for each sequence, quality control and length trimming for the raw reads were needed. The paired-end reads obtained by sequencing were divided into six groups according to their unique barcodes and truncated by cutting off the barcodes and primer sequences. The remaining reads of each sample were then assembled to generate raw tags [20]; quality filtering on the raw tags was performed to obtain high-quality clean tags [21,22]. Clean tags were compared with the reference database to detect and remove chimera sequences to generate tags [23,24]. Finally, the effective tags were obtained.

Uparse (Uparse v7.0.1001, http://drive5.com/uparse/) [25] was used to cluster all of the effective tags. The effective tags with ≥97% identity were clustered into the same operational taxonomic unit (out). The OTUs with the highest frequencies were selected as representatives of the OTU sequences. We removed OTUs with only one sequence from the dataset, since these unique OTUs could result from sequencing errors. The representative sequence for each OTU was annotated by the GreenGene database based on the RDP classifier, and multiple sequence alignment was performed by MUSCLE software [26,27,28].

Alpha diversity and beta diversity analyses; the observed species, including chao1; the Shannon index; the Simpson index; abundance-based coverage estimator (ACE); good-coverage; rarefaction analysis; rank abundance analysis; principal component analysis (PCA); principal coordinate analysis (PCoA); unweighted pair-group method with arithmetic means (UPGMA); nonmetric multi-dimensional scaling (NMDS) analysis; and T-test analysis were performed by QIIME and displayed with R software [22].

## 3. Results

### 3.1. Sequencing Results

Illumina Miseq sequencing generated a total of 434,468 raw tags representing five samples, with individual reads ranging from 81,329 to 92,592 bp. After quality control, the remaining high-quality reads in the dataset, with an average of 419 bp, were presented. After qualification and removal of chimeras from raw tags, 313,044 effective tags were finally obtained by HTS. The Q20 values were from 98.24 to 98.41, indicating that the databases were of high quality (Table 2).

**Figure 1:**
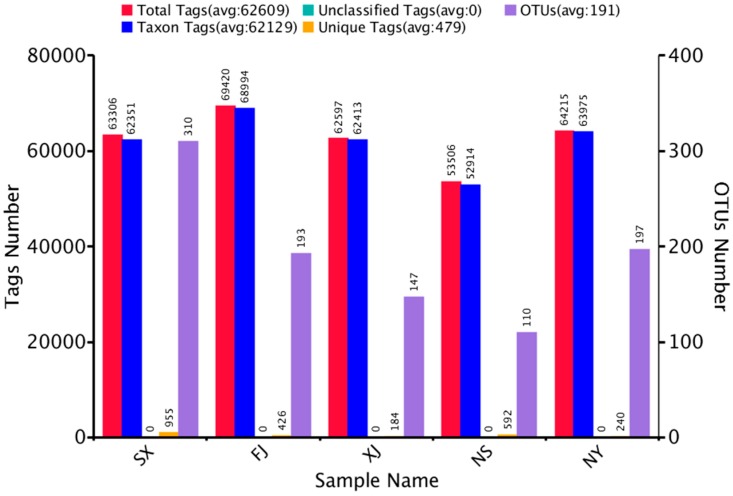
Operational taxonomic unit (OTU) clustering and annotation statistics of each sample.

In order to study the species diversity of the sample, the effective tags of samples were grouped into OTUs based on 97% identity. As shown in Figure 1, after removing singletons, the number of valid OTUs was 957, with an average of 62,129 sequences of annotated information.

**Figure 2:**
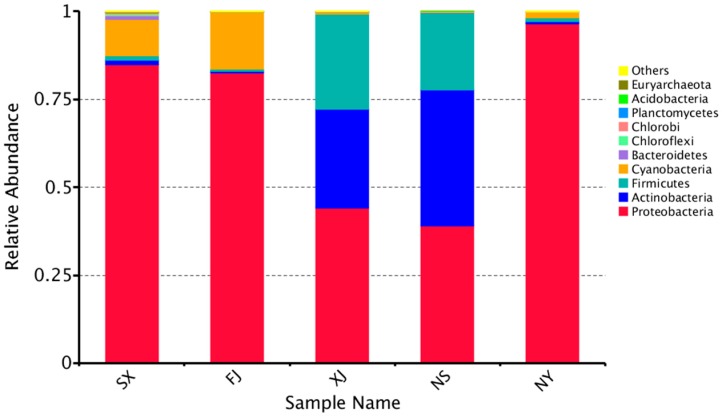
Relative abundance of the top 10 bacteria at the phylum level of taxonomy. The other phyla are included as “Others.”

The top 10 microorganism populations from five samples were enumerated. The 10 largest phyla are shown in Figure 2. Proteobacteria dominated the observed sequences at the phylum level, representing 84.8%, 82.5%, 44.1%, 96.5%, and 39.1% of the total number of species in SX, FJ, XJ, NY, and NS, respectively. In addition, Actinobacteria were found to be the predominant phylum in NS (38.56%) and XJ (28.15%). Meanwhile, Firmicutes were also high in the NS and XJ samples, accounting for 27.00% and 21.89%, respectively. This was followed by Cyanobacteria, which accounted for 16.29% in FJ.

**Figure 3:**
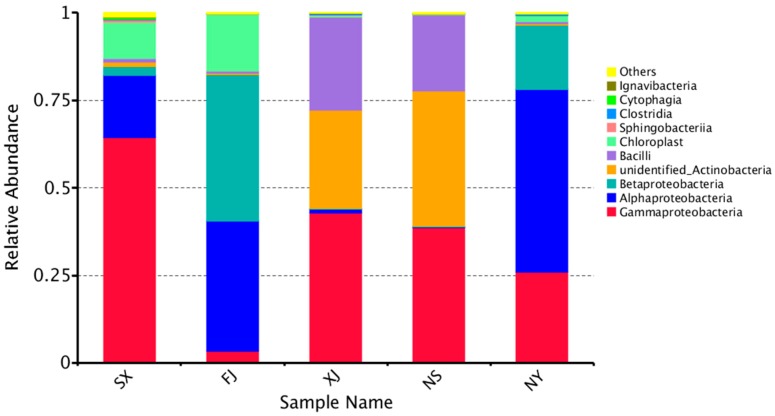
Relative abundance of the top 10 bacteria at the class level of taxonomy. The other classes are included as “Others.”

Within Proteobacteria, Alphaproteobacteria was the most dominant group (52.23%) at the class level in the NY sample. Gammaproteobacteria occupied a large part of the relative abundance in SX (64.57%), XJ (42.97%), and NS (38.58%), respectively (Figure 3). Betaproteobacteria were high in FJ (41.90%) (Figure 3).

**Figure 4:**
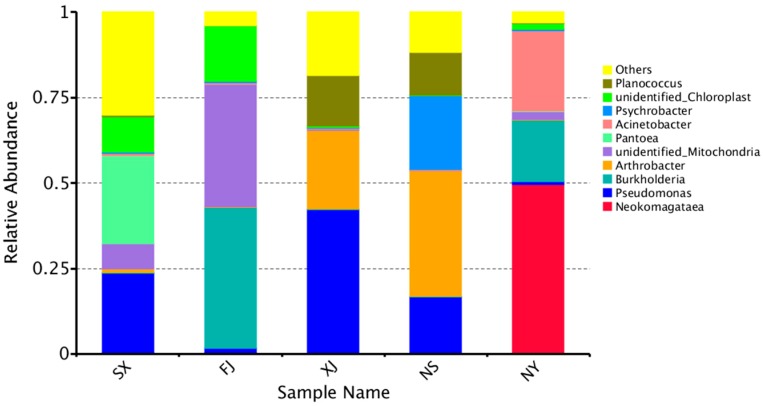
Relative abundance of the top 10 bacteria at the genera level of taxonomy. The other genera are included as “Others.”

In terms of genera, *Pantoea* (25.69%) was more abundant than other genera, followed by *Pseudomonas* (23.25%) in SX. *Burkholderia* (41.22%) was found to be the most dominant in FJ. *Pseudomonas* (41.99%), followed by *Arthrobacter* (23.03%) was most dominant in XJ; *Arthrobacter* (36.87%) was the most dominant, followed by *Psychrobacter* (21.39%) in NS; and *Neokomagataea* (49.64%) was the most dominant, followed by *Acinetobacter* (23.56%), in NY. However, a much higher proportion of unidentified sequences existed in all samples (Figure 4).

**Figure 5:**
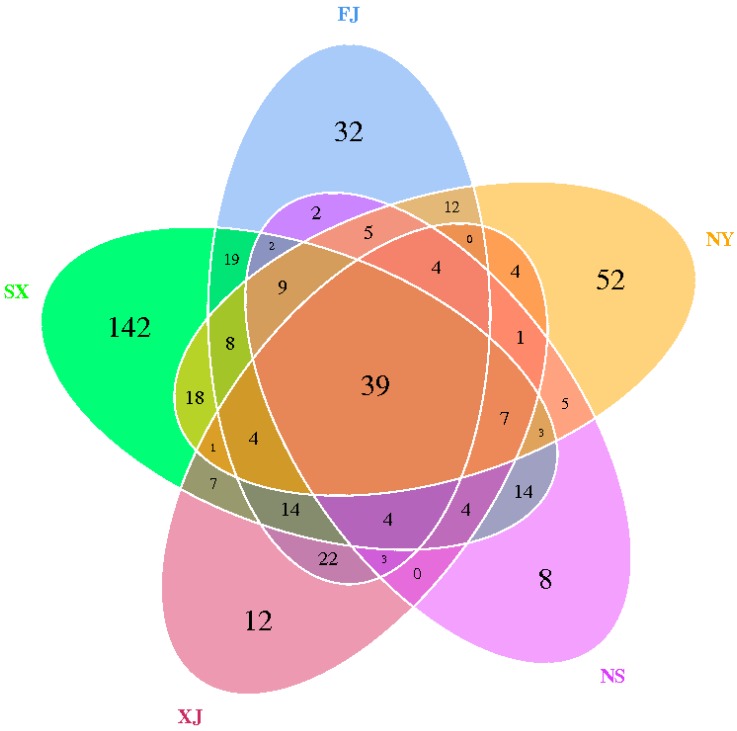
Venn graphs of the five *P. sinese* samples. The numbers inside the diagram indicate the numbers of OTUs.

We compared the corresponding number of OTUs and bacterial compositions in different samples. The comparison of the OTUs of the different samples at different eco-regions, as indicated by Venn graphs, were used to show the shared and unique OTUs in different samples. The comparison of the OTUs of the different samples indicated that the five samples shared 39 common OTUs and that samples SX, FJ, NY, NS, and XJ had 142, 32, 52, 8, and 12 unique OTUs, respectively. In addition, even the samples within the same province, NY (99), had higher unique OTU numbers than NS (37) (Figure 5).

### 3.2. Alpha Diversity Analysis

**Figure 6:**
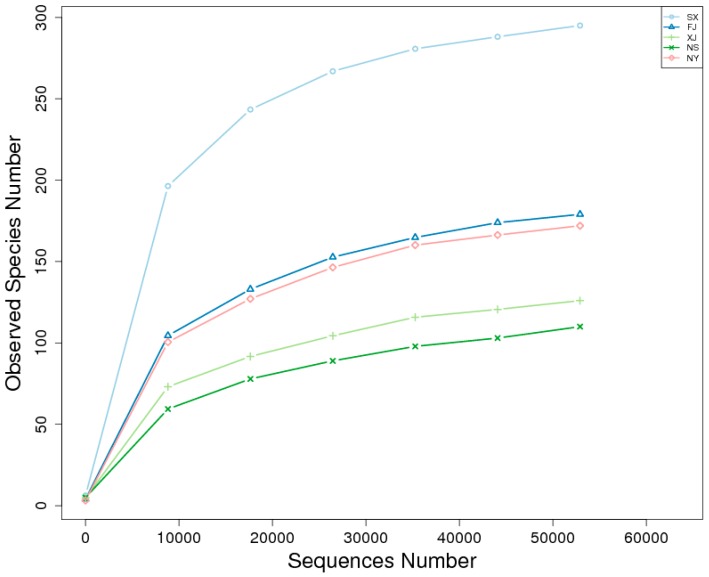
Rarefaction curves of bacterial community composition in five samples.

The trend of rarefaction curves suggested that there was sufficient sampling of the microbial communities and indicated that each sample was different (Figure 6). Good’s coverage estimator values ranged from 99.9% to 100% (Table 3), indicating that the sequence numbers per sample were high enough to capture the majority of the 16S rRNA gene sequences to show bacterial diversity.

The alpha diversity parameters of each sample are displayed in Table 3. The observed species were highest in the SX sample at 295 and lowest in the NS sample at 110. Moreover, the Shannon index of the SX sample was the highest (3.795). In contrast, that of the FJ sample was the lowest (2.165). We found that SX had the highest Chao1 (308.097), ACE (309.216), and PD_Whole Tree indices (38.881), while the NS sample had the lowest Chao1 (126.240), ACE (145.058), and PD_whole_tree indices (10.837), respectively. The Simpson indices of diversity parameters proved to be the lowest and varied from 0.677 to 0.863 in all samples.

### 3.3. Beta Diversity Analysis

**Figure 7:**
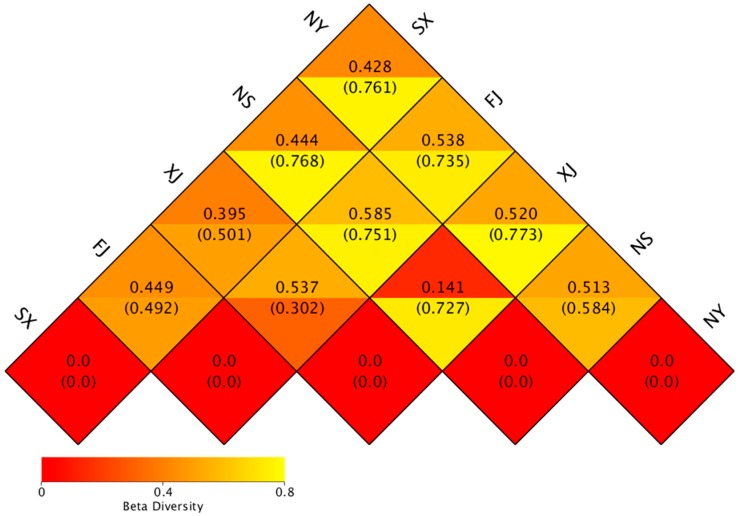
Heat map of the Beta diversity index for all samples.

A heat map of the Beta diversity index was constructed (Figure 7). The results revealed that the samples collected from FJ shared highest level correlation rates of species with other sampling sites: 0.538 in NY, 0.585 in NS, 0.537 in XJ, and 0.449 in SX, respectively.

**Figure 8:**
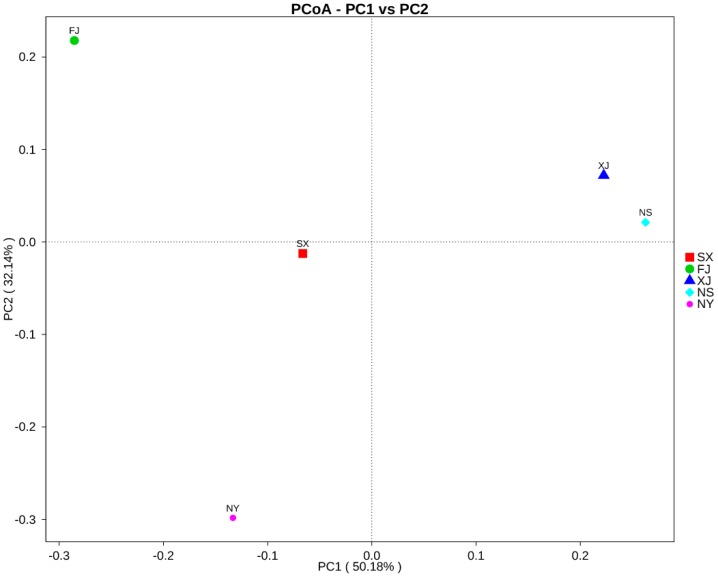
Principal coordinate analysis (PCoA) plot based on unweighted Unifrac metrics for all samples.

**Figure 9:**
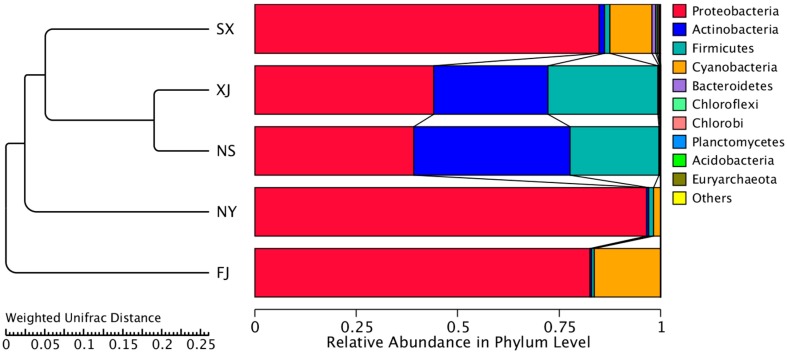
UPGMA clustering tree based on the weighted unifrac distance. The relative abundances of the top ten phyla in all samples are indicated, and the rest of the phyla are indicated as “Others.”

Meanwhile, the principal coordinate analysis (PCoA), the unweighted pair-group method with arithmetic (UPGMA), and the canonical correlation analysis (CCA) were performed to visualize and compare the relationships of the microbial communities among different samples. The results of the PCoA based on unweighted Unifrac distances demonstrated that XJ and NS samples tended to cluster together according to PC1 (50.18%) and PC2 (32.14%), representing a strong separation based on the different samples (Figure 8). For the diversity analysis, a UPGMA tree was constructed, and the results showed that samples from XJ and NS were clustered together. Moreover, they and SX clustered separately as compared to other samples (FJ and NY). The results of the UPGMA clustering tree confirmed those of PCoA. At the phylum level, FJ contained the lowest abundances of Actinobacteria and Firmicutes, but the highest abundance of Proteobacteria (Figure 9).

**Figure 10:**
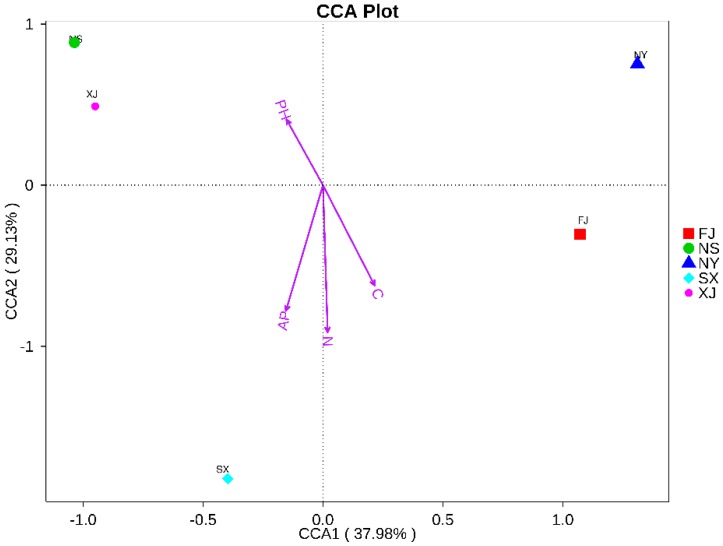
Canonical correlation analysis (CCA) analysis for all samples.

The canonical correlation analysis (CCA) indicated that the total nitrogen content was the major factor contributing to the differences between the endophytic bacterial communities and environmental factors. The first ordination axis was strongly correlated with the soil effective phosphorus and total carbon and nitrogen contents and explained 37.98% of the total variability. The second ordination axis was unrestricted (29.13% of the contribution rate) and was mainly associated with pH. According to results of the CCA analysis, the soil total carbon content, total nitrogen, effective phosphorus, and pH were the major factors explaining the variations in the overall structure in the study (Figure 10).

T-tests were used to reveal statistically significant different species (*p* < 0.05) in different samples at distinct taxonomy levels. As a result, significant differences were found in the bacterial community composition among all sampling locations, except between NY and XJ (*p* = 0.045). The effect of the sample origin was significant.

## 4. Discussion

The information gathered in this study provides a baseline of information on the composition of endophytic microbial communities in *P. sinese* roots in five samples. In addition, this information could provide a starting point for future investigations directed toward developing a better understanding of the role of each member within these microbial communities and optimizing plant growth promotion for endophytic microbial communities with the aim of improving production and quality.

In the current study, it was suggested that endophytic fungi provide essential nutrients for their hosts’ growth and defend hosts from biotic and abiotic stresses. In return, the host plant alters the composition of the microbial community to a large extent [4,5,6,7,8,9]. Although several investigations have already revealed many important aspects of *P. sinese* endophytic bacteria [10,11,12], little information exists about indispensable functions in *P. sinese.*

In our study, we surveyed the endophytic bacteria composition and diversity in *P. sinese* based on the high-throughput sequencing method, which can provide a large amount of data with high accuracy and a low cost. Many endophytic bacteria were found to exist in the roots associated with *P. sinese*. A total of 313,044 effective sequences and 957 OTUs were yielded from five samples. The geographic conditions have a certain impact on the endophytic bacteria diversity among *P. sinese* from different sampled sites.

*P. sinese*, used as a feedstock in graziery and agroforestry for biomass production, reforestation, or site restoration, was introduced from FJ into other sites in recent years. We found that a given plant genotype apparently selects a particular microbiome, and the structure of endophytic bacteria is correlated with the host plant. For example, Proteobacteria was the dominant phylum in all samples, followed by Actinobacteria, Firmicutes, and Cyanobacteria. This result agrees with a previous study; these phyla present in many environments [29,30]. Moreover, these results agree with those obtained by Lin et al. (2018) [31], who detected that Proteobacteria was the main phylum. Gammaproteobacteria and Alphaproteobacteria were the two main classes within Proteobacteria. Findings from [30,32] were similar; these classes were also found in some plant species. Cyanobacteria are ubiquitous microorganisms and constitute a high portion of soil microbes [33]. The genera Pantoea, Pseudomonas, Burkholderia, Arthrobacter, Psychrobacter, and Neokomagataea were significantly dominant in five samples. Furthermore, in agreement with a previous study [34], these *P. sinese* root-associated microbes are beneficial to the plants.

Additionally, for the endophytic bacteria diversity analysis in *P. sinese*, Lin et al. [31] screened and analyzed the dynamic endophytic bacteria in roots, stems, and leaves at different growth stages of *P. sinese.* The results revealed various diversities of endophytic bacteria in *P. sinese* and found that *Ralstonia* and *Lactococcus* were dominant at the genus level. However, in our study, *Pantoea*, *Pseudomonas*, *Burkholderia*, *Arthrobacter*, and *Neokomagataea* were the most dominant genera. This difference may be a result of different methods having different detection capabilities for endophytic bacteria. The results of our study indicate that these top genera may play pivotal roles in maintaining and shaping the structures and functions of bacterial communities in *P. sinese* (the results were not published).

On the other hand, evidence was found that the strains restricted to an ecological niche generally hold genetic characteristics and delineate according to their geographical origins [35]. Study of this phenomenon could give important information about the abundance of bacterial species on the planet and their ecological roles. In our present study, the endophytic bacteria structure was different among the five samples. Interestingly, samples from SX contained the most unique OTUs. The genera *Pantoea* and *Pseudomonas* were mainly present in samples from SX, *Burkholderia* in FJ, *Pseudomonas* and *Arthrobacter* in XJ, *Arthrobacter* and *Psychrobacter* in NS, and *Neokomagataea* and *Acinetobacter* in NY. Thus, different ecological types of endophytic bacteria exist in distinct geographic regions, and this should be an important consideration for the selection of endophytic bacteria inoculation. Although NY accounted for a lower proportion of the Shannon index, it contained 20.58% OTUs—more than the other samples—due to its different environmental factors. Additionally, compared to the other sampled sites, we found that all of the alpha indices of the SX sample were higher, suggesting that the SX sample possessed a higher bacterial richness and diversity. SX contains over 17.7 times more unique OTUs than NS. In addition, the Venn graphs of the five samples supported this conclusion. The heatmaps of the PCoA, UPGMA, and CCA analyses evidently demonstrated that the bacterial diversity was different among the five samples and found that the particular environmental factors affected variation among microbial communities.

In general, our results demonstrated clear differences in the relative abundances of certain species among the five samples examined, suggesting that some endophyte species may preferentially proliferate in a certain eco-region and play ecological roles that are distinct from those of other endophytes.

Previously, most researchers [36,37,38,39] demonstrated that both the abiotic conditions (temperature, soil pH, rainfall, etc.) and biotic conditions (genotypes of host plants and their distribution) might affect the diversity and composition of the endophytic bacteria species. Consistent with these reports, our results showed that the endophytic bacteria communities can be grouped into two bigger ecological regions (Fujian province and the northwest region) according to their geographic origins (Figure 9). The high correlation between the geographic regions and the bacterial genotypes may be attributed to the different environmental factors and the soil characteristics of the sampling sites. The physical and chemical properties of the soil, such as soil texture, mineral composition, and organic matter, affect the community structure of endophytic bacteria in plants [40]. Our results are similar to the results obtained by this study. We found that the majority of genotypes were associated with a certain geographic location (Figure 4); this might be explained by the fact that these sites had diverse soil characteristics. The content of available phosphorus in soil physico-chemical factors of SX samples was significantly higher than that in the other four soil samples. The CCA analysis showed that the endophytic bacterial communities of the SX samples were mainly affected by the content of available phosphorus in the soil. This indicates that the content of available phosphorus in soil had a certain effect on the structure of the endophytic bacteria community in the roots of *P. sinese*.

Additionally, previous studies have demonstrated that the physical and chemical properties of soil change with a decrease in soil fertility and that this is associated with a decrease in microbial community abundance and diversity [40,41]. In contrast to other sites, the lowest abundance of endophytes was detected in the NY sample due to its minimal Shannon index. Similarly, the Chao1 index was the lowest in the NS sample, which may be related to the soil factors in the growing area. Moreover, our results showed that the total nitrogen, available phosphorus, and total carbon content in sandy land and saline-alkali land are obviously lower in NY compared with the other three samples; this unique property is correlated with the genotypes, as the NY formed a single distinct group in the cluster (Figure 9). These results are consistent with earlier reports and demonstrate the fact that the soil characteristics might be more important than the climate factors in the determination of bacterial diversity or community composition.

Overall, our results imply that the bacterial community might be determined by the geographic origin. Furthermore, these results also support the hypothesis that soil properties and climate factors may drive the structure and composition of bacterial populations. The living environment was the major factor contributing to the difference; the relationship between endophytic bacterial diversity and the others environmental factors, such as saline-alkali and light, needs further study.

In conclusion, from the alpha diversity analysis and beta diversity analysis, many endophytic bacteria were found in the roots of *P. sinese* from the five samples. The endophytic bacterial structure and composition differed in different samples, and the geographic conditions and climate factors had certain impacts on the endophytic bacterial diversity and abundance of *P. sinese* from the different samples. Further studies on the roles of these endophytic bacteria are required for characterization. This study might be useful for growth improvement and might be useful for improving the production and quality of *P. sinese*.

## 5. Conclusions

It is essential to investigate the endophytic bacterial diversity in the roots of *P. sinese.* In this study, the composition diversity and differences in endophytic bacteria in roots in different growth eco-regions associated with *P. sinese* were analyzed using high throughput sequencing technology. Similar to many studies, we found that Proteobacteria was the most abundant phylum in all samples; Gammaproteobacteria and Alphaproteobacteria accounted were dominant at the class level in five samples, indicating that there is host selection of host-specific endophytes in host species in distinct eco-regions. However, our investigation also revealed the compositions of the endophytic bacterial communities, and the diversity was distinctly different among these samples. The different soil characteristics can provide an important contribution to the understanding of their effects on the bacterial communities associated with *P. sinese.* Our results demonstrated that *P. sinese* modulates the bacterial microbiota composition by recruiting specific endophytic bacteria, which may help to improve its protection and growth, and further provides new opportunities for exploring their potential utilization. In this study, we collected samples from only five sites without considering the dynamic population and other factors. In the long run, more tissues and environmental factors should be covered to better study the varieties and functions of the endophytic bacterial community to improve *P. sinese* growth and productivity.

## Figures and Tables

**Table 1:** Geographical sources and physicochemical properties of soil samples.

Sampling Site	SX	FJ	XJ	NS	NY
	Yanan, Shaanxi Province	Fuzhou, Fujian Province	Jichang Xinjiang Uygur Autonomous Prefecture	Bayannaoer, Inner Mongolia (sandy land)	Bayannaoer, Inner Mongolia (saline-alkali land)
Altitude	1000 m	600 m	560 m	1500 m	1500 m
Latitude and longitude	36°63′97″ N, 109°32′07″ E	26°08′55″ N, 119°28′22″ E	44°04′21″ N, 87°18′19″ E	40°13′55″ N, 107°05′05″ E	40°30′19″ N, 107°02′98″ E
Annual average temperature (°C)	13.0	19.0	6.0	4.0	4.0
Annual average precipitation (mm)	576.9	1700.0	150.0	50.0	50.0
Total nitrogen (%)	0.62	0.53	0.24	0.12	0.04
Total phosphorus (mg/kg)	1784	2065	2036	1465	1718
Effective phosphorus (mg/kg)	12.6	10.0	10.8	3.0	3.5
Available potassium (mg/kg)	132.9	112.8	324.7	154.1	99.5
Total carbon (%)	2.47	4.00	1.44	0.77	0.23
pH	8.35	6.98	8.27	8.9	9.38

**Table 2:** Sequencing results from five samples.

Raw Tags	Clean Tags	Effective Tags	Base (nt)	AvgLen (nt)	Q20	GC%	Effective%
89,003	67,460	63,306	26,672,103	421	98.24	54.11	71.13
92,592	70,360	69,420	28,879,554	416	98.28	55.08	74.97
88,774	69,217	62,597	26,450,228	423	98.33	53.25	70.51
81,329	64,091	53,506	22,509,234	421	98.35	53.55	65.79
82,770	65,669	64,215	26,657,088	415	98.41	53.72	77.58

**Table 3:** Median alpha diversity indices.

Sample Name	Observed Species	Shannon	Simpson	Chao1	ACE	Goods Coverage	PD_Whole Tree
SX	295	3.795	0.863	308.097	309.216	0.999	38.881
FJ	179	2.165	0.677	192.286	200.258	0.999	28.621
XJ	126	2.982	0.816	140.130	145.552	1.000	23.883
NS	110	2.780	0.787	126.240	145.058	0.999	10.837
NY	172	2.161	0.665	182.800	190.536	0.999	16.980
